# Generalisability of an adult-trained AI model to paediatric coeliac disease diagnosis: a multicentre external validation study

**DOI:** 10.1016/j.eclinm.2026.104246

**Published:** 2026-10-12

**Authors:** Florian Jaeckle, Peter M. Gillett, Kathryn J. Kirkwood, Shonali Natu, James Y.H. Chan, Adrian C. Bateman, Mark J. Arends, Elizabeth J. Soilleux

**Affiliations:** a Department of Pathology, University of Cambridge, Cambridge, UK; b Lyzeum, Cambridge, UK; c Department of Child Life and Health, University of Edinburgh, Royal Hospital for Children and Young People, UK; d Department of Pathology, Western General Hospital, Edinburgh, UK; e University Hospital of North Tees, North Tees and Hartlepool NHS Foundation Trust, Stockton-on-Tees, UK; f Cambridge University Hospitals NHS Foundation Trust, Cambridge, UK; g University Hospital Southampton NHS Foundation Trust, Southampton, UK; h Edinburgh Pathology & Centre for Comparative Pathology, Institute of Genetics & Cancer, University of Edinburgh, Edinburgh, UK

**Keywords:** Coeliac disease, Machine learning, Paediatrics, Digital pathology

## Abstract

**Background:**

Coeliac disease diagnosis on duodenal biopsies is limited by interobserver variability. We have previously demonstrated pathologist-level performance with our artificial intelligence (AI) model for the histopathological diagnosis of adult coeliac disease, but not in paediatric practice. As paediatric coeliac disease screening programmes expand internationally, accurate and scalable diagnostic tools are needed. We investigated whether an AI model trained exclusively on adult whole-slide images (WSIs) can generalise to paediatric coeliac disease diagnosis across independent centres.

**Methods:**

A training and validation dataset of 9958 WSIs from 8421 adult patients (961 coeliac disease; collected Jan 1, 2014, to March 31, 2025) from five centres was used to develop an ensemble of multiple-instance learning models using features from a foundation model. Testing was performed on 708 consecutive paediatric patients (86 with coeliac disease) from two centres (Edinburgh and Southampton) not included in training. Model calibration was assessed, and probability outputs were grouped into clinically interpretable categories.

**Findings:**

In adult cross-validation, the AI model had an area under the receiver operating characteristic curve (AUC) of 99.1% (95% CI 98.9–99.4), sensitivity of 89.3% (87.2–91.1), specificity of 98.8% (98.5–99.0), and negative predictive value (NPV) of 98.6% (98.3–98.9). On testing (paediatric) datasets, performance remained high (AUC 98.8%, 95% CI 98.2–99.4; sensitivity 80.2%, 70.6–87.3; specificity 98.4%, 97.1–99.1; NPV 97.3%, 95.7–98.3). Restricting analysis to predictions outside the intermediate-probability range (predicted coeliac disease probability <10% or ≥65%; 604 [85.3%] of 708 cases) improved sensitivity to 100% (95% CI 93.7–100.0) and specificity to 98.7% (97.4–99.4). No misclassifications were observed among high-confidence predictions (<2% or ≥85%; 467 [66.0%] of 708 cases). The expected calibration error was 0.03. Performance improved significantly when biopsies from both duodenal sites (bulb [D1] and descending [D2/3]) were considered.

**Interpretation:**

Our AI model, trained on adult biopsies, generalises to paediatric coeliac disease diagnosis across centres and scanner platforms. Well calibrated probability outputs provide clinically interpretable measures of diagnostic confidence and could support safe identification of coeliac disease-negative biopsies within defined thresholds. These findings demonstrate the feasibility of applying adult-derived AI models in paediatric populations and reinforce the importance of multi-site (D1 & D2) biopsy sampling.

**Funding:**

The National Institute for Health and Care Research.

**Research in context**

**Evidence before this study**

We searched Google Scholar from database inception to Jan 31, 2026, using terms related to “coeliac disease”, “celiac disease”, “artificial intelligence”, “deep learning”, “machine learning”, “paediatric”, and “children”. Reference lists of relevant articles were also screened.

Previous studies have shown that AI models can achieve high diagnostic accuracy for coeliac disease on duodenal biopsy images, often approaching pathologist-level performance. However, these studies have largely focused on adult populations, with limited external validation and little assessment of model calibration. Evidence in paediatric populations remains scarce, despite increasing clinical demand from screening programmes. To our knowledge, no study has yet externally validated a machine learning model for coeliac disease diagnosis specifically in a paediatric population, leaving a critical clinical gap despite increasing demand from paediatric screening programmes.

**Added value of this study**

This study demonstrates that a deep learning model trained exclusively on adult duodenal biopsy whole-slide images can generalise effectively to paediatric coeliac disease diagnosis across independent centres and scanner platforms. We show that well-calibrated probability outputs enable clinically interpretable levels of diagnostic confidence, with no misclassifications observed among high-confidence predictions. Additionally, we provide quantitative evidence that combining biopsies from both the duodenal bulb and descending duodenum improves diagnostic performance compared with either site alone in an AI-assisted setting.

**Implications of all the available evidence**

Taken together with existing literature, these findings support the potential for AI-based tools to assist histopathological assessment of coeliac disease in children, particularly in the context of increasing demand from screening programmes. Our results reinforce current guideline recommendations for multi-site duodenal biopsy sampling and highlight the importance of probability calibration for safe clinical deployment of AI models. More broadly, this work supports the feasibility of leveraging adult-derived datasets to develop AI tools for paediatric applications, which may help address the current underrepresentation of children in medical AI research. Future studies should focus on prospective validation in routine workflows, evaluation of clinical impact, and assessment across more diverse populations.

## Introduction

Coeliac disease is a common autoimmune disorder triggered by gluten ingestion, with a global seroprevalence of 1.4% and a biopsy-confirmed prevalence of 0.7%,^1^ but the majority of cases remain undiagnosed.^2^ Symptoms range from classical features of malabsorption, including chronic diarrhoea, weight loss, anaemia, and fatigue, to extra-intestinal manifestations such as dermatitis herpetiformis and infertility.^3^ If left untreated, coeliac disease carries a significantly elevated risk of serious long-term complications, including lymphoma and adenocarcinoma.^3^^,^^4^ Beyond these physical manifestations, coeliac disease particularly in children and adolescents is increasingly recognised to carry substantial psychosocial burden, including anxiety, food-related hypervigilance, social restriction, and impaired quality of life associated with maintaining a strict gluten-free diet,^5^^,^^6^ highlighting the importance of an accurate diagnosis.

The gold standard for diagnosis, as recommended by NICE, is histological examination of duodenal biopsies,^7^ however, this process is subject to significant interobserver variability as acknowledged by the recent European Society guidelines.^8^ The mean probability of two pathologists agreeing on a coeliac disease diagnosis from digitised whole-slide images has been shown to be as low as 0.73, with a Cohen's kappa (κ) of 0.59 in the absence of clinical or laboratory data, improving only modestly to 0.80 when serological information is included.^9^ Earlier studies using different methodological approaches have also demonstrated substantial variability in histopathological assessment: Corazza and colleagues^10^ reported an overall κ of 0.35 using the Marsh–Oberhuber classification, improving to 0.55 with the simplified Corazza–Villanacci classification, while Arguelles-Grande et al.^11^ reported κ values ranging from 0.419 to 0.888 across different pathology practice settings. This diagnostic inconsistency has significant implications for patient care and underscores the need for more objective and reproducible methods of biopsy assessment.

Advances in artificial intelligence (AI) have shown considerable promise in addressing this challenge. In our previous multicentre study, we demonstrated that our AI models achieve expert pathologists-level accuracy in detecting coeliac disease from duodenal biopsies in adult patients.^12^ Although multiple studies have explored AI-based approaches to coeliac disease diagnosis,^12^, ^13^, ^14^, ^15^, ^16^ as summarised in the recent review by Mahmoudi Ghehsareh and colleagues,^17^ the paediatric domain remains underexplored. Syed et al.^18^ attempted to address this gap by developing a convolutional neural network to classify coeliac disease, environmental enteropathy, and normal duodenal biopsies in children; however, the study has a critical methodological limitation: environmental enteropathy images were sourced from hospitals in Pakistan and Zambia, whereas coeliac disease and normal images came from a different institution in the USA, and the two groups were acquired using different imaging methods. As the disease label and image source are therefore entirely confounded, it is impossible to determine whether the proposed model learned to identify pathological features or simply to recognise the origin of the images. To our knowledge, no study to date has demonstrated pathologist-level AI performance specifically in paediatric coeliac disease biopsies. Furthermore, some authors have suggested that paediatric and adult coeliac disease may differ in their biological and histopathological characteristics, with reported differences in gene expression^19^ as well as in clinical presentation and mucosal pathology across age groups.^20^, ^21^, ^22^, ^23^

There is an increasing gap between the amount of AI research in paediatric coeliac disease compared to adult populations. Mass screening strategies for paediatric coeliac disease have been reported across 17 countries,^24^ most notably in Italy, which has become the first country to mandate nationwide screening for coeliac disease in children. A pilot programme has screened over 5000 children aged 2–3, 6–7, and 10–11 years across four regions,^25^ using serological testing for coeliac-specific autoantibodies, including anti-tissue transglutaminase IgA with concurrent assessment of total IgA. Children with positive screening results are referred for specialist evaluation, where diagnosis is confirmed according to established paediatric guidelines through repeat serology and, in cases not meeting serology-based diagnostic criteria (e.g., anti-tissue transglutaminase IgA levels below ten times the upper limit of normal^26^), duodenal biopsy sampling from both the bulb (D1) and descending duodenum (D2), as recommend by the 2020 European Society Paediatric Gastroenterology, Hepatology and Nutrition (ESPGHAN) Guidelines for Diagnosing Coeliac Disease.^26^ This multi-site approach is particularly important given the recognised existence of ultra-short coeliac disease, in which villous atrophy is confined to the duodenal bulb and may be missed by descending duodenal sampling alone.^27^^,^^28^ As programmes such as these expand internationally, the number of children undergoing confirmatory diagnostic assessment, including histological evaluation, will increase, driving demand for accurate, scalable tools to support coeliac disease diagnosis on paediatric biopsies.

Despite this growing clinical need, children remain profoundly underrepresented in medical AI development. A cross-sectional analysis of 876 FDA-authorised AI and machine learning-enabled medical devices found that only 17% were explicitly labelled for use in children, and of those, only one in five had reported development using paediatric datasets.^29^ A separate review of AI medical devices in radiology found that only 4 of 213 devices were clearly labelled for paediatric use.^30^ This regulatory gap exposes children to the risk of off-label use of tools validated only on adult populations, a concern that extends well beyond radiology. Children are not simply small adults: physiological, anatomical, and histological differences mean that AI tools trained exclusively on adult data may fail or perform sub-optimally when applied to paediatric cases, a risk that remains poorly characterised for the vast majority of deployed tools.

Against this backdrop, we investigate whether an AI model trained exclusively on adult whole-slide images (WSIs) can generalise to paediatric coeliac disease diagnosis across unseen clinical centres, and whether key histomorphological features of coeliac disease are sufficiently conserved across age groups to permit this transfer of learning.

## Methods

### Dataset and study cohorts

The training and validation dataset comprised haematoxylin and eosin-stained duodenal biopsy whole-slide images from 8421 adult patients (961 coeliac disease, 6262 normal, and 1198 with another diagnosis; ≥18 years old) collected between Jan 1, 2014, and March 31, 2025, from five hospitals, scanned at 40 × magnification (∼0.25 μm per pixel) using five different scanners from four manufacturers. Most contributing centres provided a series of consecutive biopsies, after which datasets were enriched for coeliac disease cases and, in some instances, for other non-coeliac pathological abnormalities to ensure adequate representation of diagnostically relevant classes (Table 1). Supplementary Table S1 in Supplementary Material S1 contains a detailed breakdown of the non-coeliac, but pathological, diagnoses.

**Table 1:** Training and testing datasets.

	Training data			Testing data		
	Coeliac (n = 961)	Normal (n = 6262)	Other (n = 1198)	Coeliac (n = 86)	Normal (n = 479)	Other (n = 143)
Source						
Addenbrooke’s	201 (21%)	3228 (52%)	777 (65%)	–	–	–
North Tees	16 (2%)	1517 (24%)	172 (14%)	–	–	–
Heartlands	633 (66%)	563 (9%)	0 (0%)	–	–	–
Glasgow	70 (7%)	538 (9%)	34 (3%)	–	–	–
UHCW	41 (4%)	416 (7%)	215 (18%)	–	–	–
Edinburgh	–	–	–	55 (64%)	159 (33%)	23 (16%)
Southampton	–	–	–	31 (36%)	320 67 (%)	120 (84%)
Scanner						
Leica Aperio AT2	273 (28%)	1008 (16%)	181 (15%)	–	–	–
Hamamatsu Nanozoomer XR	194 (20%)	187 (3%)	0 (0%)	–	–	–
Hamamatsu Photonics K.K.	16 (2%)	1517 (24%)	172 (14%)	–	–	–
Roche Ventana iScan HT	245 (25%)	189 (3%)	0 (0%)	–	–	–
Philips IntelliSite Ultra Fast Scanner	233 (24%)	3361 (54%)	845 (71%)	55 (64%)	159 (33%)	23 (16%)
3DHISTECH Kft. Pannoramic 1000	–	–	–	31 (36%)	320 (67%)	120 (84%)
Sex						
Female	632 (66%)	3608 (58%)	557 (46%)	57 (66%)	229 (48%)	60 (42%)
Male	329 (34%)	2651 (42%)	640 (53%)	29 (34%)	248 (52%)	80 (56%)
Age, years						
Median (IQR)	44 (32–62)	64 (50–75)	66 (53–75)	12 (8–15)	13 (9–15)	13 (9–15)
1–5	–	–	–	10 (12%)	52 (11%)	15 (10%)
6–10	–	–	–	26 (30%)	108 (23%)	34 (24%)
11–17	–	–	–	50 (58%)	319 (67%)	94 (66%)
18–29	195 (20%)	348 (6%)	47 (4%)	–	–	–
30–39	191 (20%)	437 (7%)	89 (7%)	–	–	–
40–49	184 (19%)	727 (12%)	119 (10%)	–	–	–
50–59	116 (12%)	1017 (16%)	202 (17%)	–	–	–
60–69	146 (15%)	1363 (22%)	255 (21%)	–	–	–
70–79	82 (9%)	1524 (24%)	315 (26%)	–	–	–
80–89	46 (5%)	797 (13%)	157 (13%)	–	–	–
90–99	1	49 (1%)	14 (1%)	–	–	–

Training data were collected from five UK centres and includes adult patients only. Test data were collected from two different UK centres and include paediatric cases only. Scanner model and patient age and sex are shown for both datasets. Sex data was missing for 9 cases. “Other” diagnoses comprise 14 different conditions; see Supplementary Material S1 for a full breakdown of these diagnoses. UHCW = University Hospitals Coventry & Warwickshire.

Two independent external testing cohorts were used: one from Southampton General Hospital in England and one from Western General Hospital in Edinburgh, Scotland, providing geographic and technical heterogeneity. The Edinburgh cohort was digitised using the same scanner platform as one of the training centres, whereas the Southampton cohort was scanned using a scanner from a different manufacturer, enabling assessment of cross-scanner generalisability. The combined testing datasets contain biopsies from 708 unique paediatric patients (86 coeliac disease, 479 normal, 143 other diagnosis). The mean patient age was 13 years (range 1–17 years), and 49% of patients were female (Table 1). For the entire Edinburgh cohort and the majority of the Southampton cohort, biopsies performed for suspected coeliac disease included samples from both the first (D1) and second (D2) portions of the duodenum, in line with routine clinical practice. The test dataset comprised consecutive cases and is representative of routine clinical practice. Patients undergoing repeat biopsy on a gluten-free diet were excluded from analysis.

### Ethics

All slide scans and accompanying fully anonymised patient data were obtained with Health Research Authority Approval (reference 335,851, issued on 18 December 2023), which stated that specific patient consent was not required for the study. The study was conducted in accordance with the principles of the Declaration of Helsinki and the UK Policy Framework for Health and Social Care Research.

### Reference standard and diagnostic classification

For the training dataset, reference diagnoses were derived from the original histopathology report. Cases in which the report indicated a high-confidence diagnosis were labelled directly. Where histopathological findings were equivocal or insufficient for confident classification, additional clinical information was reviewed, including coeliac serology and independent assessment by a second pathologist, to establish a reference diagnosis.

For the testing datasets, an equivalent approach was used for the Southampton cohort. For the Edinburgh cohort, reference diagnoses were established through a multidisciplinary clinical consensus process involving paediatric gastroenterologists and pathologists. This process integrated histopathological findings, serological results, and relevant clinical information, including patient history and endoscopic findings, to determine the final clinical diagnosis. All reference diagnoses were established during routine clinical care, prior to the development of the AI model. A sensitivity analysis excluding cases in which the multidisciplinary consensus diagnosis differed from the original histopathological impression is reported in Supplementary Material S3.5.

### Image pre-processing and feature extraction

Whole-slide images underwent automated tissue detection and artefact removal using the method described by Schreiber and colleagues.^31^ Tissue regions were divided into non-overlapping 256 × 256-pixel patches at 10 × magnification. Stain variability was reduced using Macenko stain normalisation.^31^ Patch-level feature embeddings were extracted using the hibou-b foundation model,^32^ which was used as a fixed feature extractor. Full details of the software environment are provided in Supplementary Material S4.

### Model development and training

We developed the AI model using four-fold leave-one-source-out cross-validation, whereby models were trained on data from all but one contributing centre and evaluated on the held-out source, enabling assessment of robustness to institutional variation. Within each fold, one binary classifier from each of the CLAM, ABMIL, and TransMIL multiple-instance learning architectures^33^, ^34^, ^35^ was trained, and their predictions were combined as an ensemble for validation. For external testing, predictions from all 12 trained models were combined with equal weighting to form the final ensemble. No additional retraining was performed prior to evaluation on the paediatric cohort. During training, data sampling strategies were applied to address imbalances in diagnostic categories and contributing centres; full details are provided in Supplementary Material S2. Validation folds reflected the composition of the held-out source, including enriched datasets where applicable. Primary performance estimates are therefore based on the independent, unenriched paediatric testing cohorts.

### Model validation and calibration

For each trained classifier, predicted coeliac disease probabilities were recalibrated using adult validation data to improve how well predicted risks matched observed outcomes. To ensure consistency at the patient level, only the scan with the highest abnormal score per case was used for calibration (reflecting clinical practice, where the most abnormal site is typically reported). Recalibration was performed separately for each classifier before ensembling using logistic regression with inverse probability weighting to account for enrichment of coeliac disease cases in the validation dataset, thereby aligning predicted probabilities with the expected prevalence in routine clinical practice. Further details of the calibration procedure are provided in Supplementary Methods S2.

### Model inference

At inference time, the final ensemble model was applied independently to each WSI (Fig. 1). For each WSI, calibrated probabilities from all 12 classifier models were averaged with equal weighting to produce a single slide-level probability. For patients with multiple WSIs, the highest slide-level probability was used as the patient-level prediction. Ensembling reduced the frequency of extreme predicted probabilities and improved prediction stability. For calculation of binary diagnostic performance metrics a default probability threshold of 0.5 was used. This threshold was prespecified prior to evaluation of the external paediatric cohorts. The confidence thresholds below were used to create a final diagnosis.

**Fig. 1:**
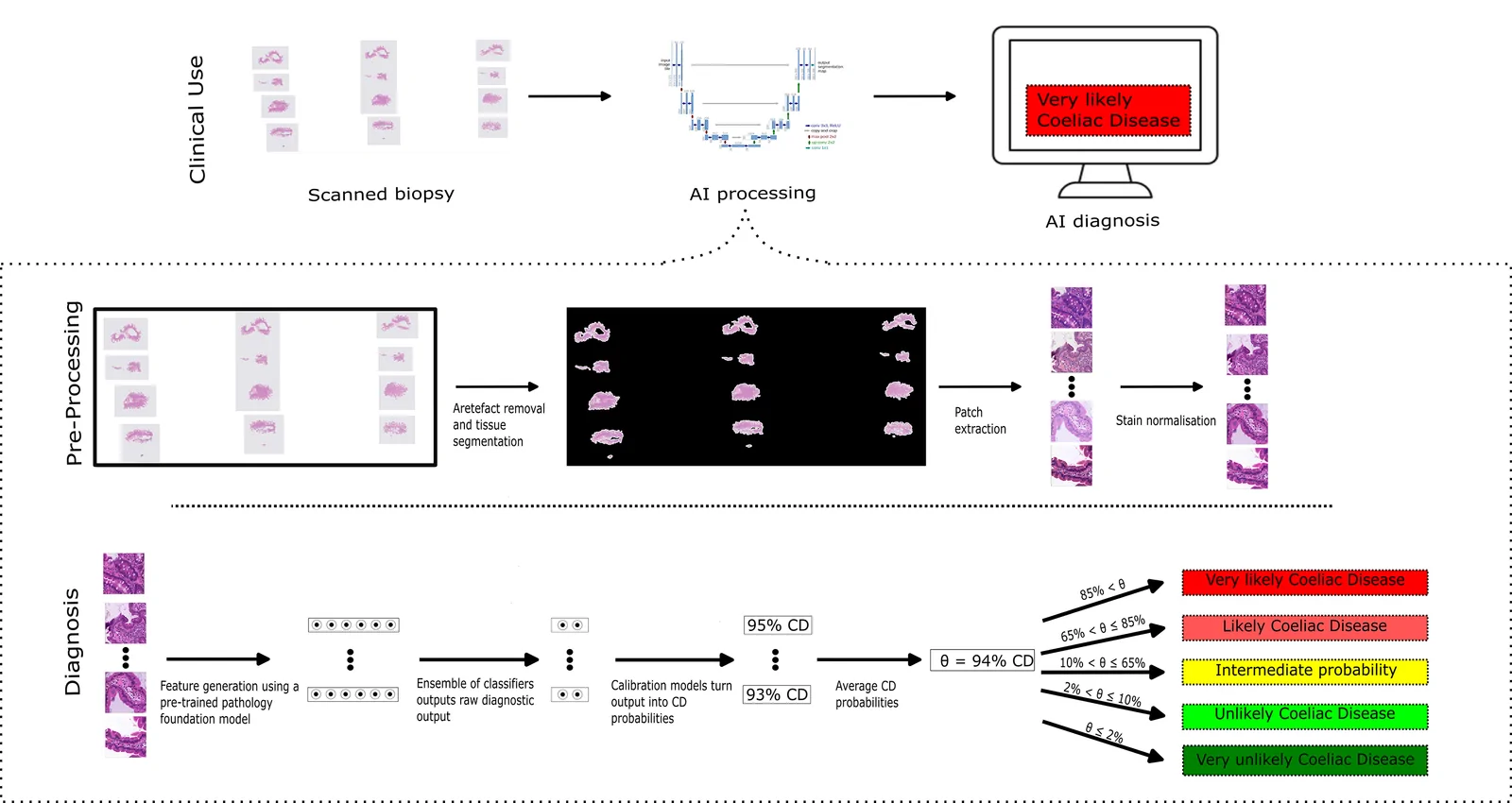
Model pipeline. The pipeline consists of the following steps: (1) digitised whole-slide images of duodenal biopsies are received; (2) artefacts are removed and tissue regions are identified; (3) tissue regions are divided into small image patches; (4) stain normalisation is applied; (5) patch-level features are extracted using a pre-trained pathology model; (6) an ensemble of classifiers generates slide-level predictions from patch-level features; (7) model outputs are calibrated to produce reliable probabilities of coeliac disease; (8) calibrated probabilities are averaged to generate a final patient-level probability; and (9) this probability is mapped to clinically interpretable diagnostic categories (very unlikely, unlikely, intermediate-probability, likely, and very likely coeliac disease). The final output is presented as a probability-informed diagnosis to support clinical decision-making. CD = coeliac disease.

### Diagnostic categories

To support clinical interpretation, predicted coeliac disease probabilities (p) were grouped into five categories using predefined confidence thresholds: very unlikely coeliac disease (p < 2%), unlikely coeliac disease (2% ≤ p < 10%), intermediate probability (10% ≤ p < 65%), likely coeliac disease (in the appropriate clinical and serological context) (65% ≤ p < 85%), and very likely coeliac disease (p ≥ 85%).

Probability thresholds were defined prior to evaluation of the external paediatric cohorts. They were selected pragmatically to favour sensitivity over specificity, reflecting the greater clinical concern associated with false-negative predictions and the intended use of the AI model as an adjunct to serological and histopathological assessment in a condition that remains underdiagnosed. Lower thresholds were aligned with typical pre-test probability in investigated populations (approximately 5–10%^36^) such that predictions below this range do not increase suspicion beyond baseline risk. Higher thresholds represent increasing levels of diagnostic confidence, with “likely” and “very likely” categories intended to support clinical decision-making in the appropriate clinical and serological context.

### Statistics

AI model performance was assessed using accuracy, sensitivity, specificity, positive and negative predictive value (PPV and NPV), and the area under the receiver operating characteristic curve (AUC) defined according to standard epidemiological conventions. Differences in AUC between AI models were compared using the DeLong test, with a two-sided p-value of less than 0.05 considered statistically significant.^37^ AI model calibration was evaluated using expected calibration error (ECE), which quantifies the agreement between predicted probabilities and observed outcome frequencies. ECE was calculated using 10 equally spaced probability bins.

### Role of funding source

The funder had no role in study design, data collection, data analysis, interpretation of results, or writing of the report.

## Results

We trained four independent AI models on a dataset of duodenal biopsies from 8421 adult patients (≥18 years) and assessed performance using leave-one-source-out cross-validation. For patients with multiple whole-slide images (WSIs), for example, biopsies obtained from both the duodenal bulb (D1) and descending duodenum (D2/3), or multiple sections prepared from the same biopsy, each WSI was analysed independently to generate a predicted probability (risk estimate) of coeliac disease. At the patient level, the maximum predicted coeliac disease probability across all WSIs was used to give the overall diagnostic classification, reflecting a clinically conservative approach in which a single highly suspicious biopsy is sufficient to support the diagnosis.

Leave-one-source-out cross-validation on our dataset of 8421 adult patients showed that our AI model achieved an overall validation accuracy of 97.7% (95% CI 97.4–98.0), sensitivity of 89.3% (87.2–91.1), and specificity of 98.8% (98.5–99.0) across all cases. Restricting analysis to high-confidence predictions (predicted coeliac disease probability p < 2% or p ≥ 85%; 7155 (85.0%) of 8421 cases) improved performance to 99.5% (95% CI 99.3–99.6) accuracy, 98.3% (97.0–99.1) sensitivity, and 99.6% (99.4–99.7) specificity (Supplementary Table S2 in Supplementary Material S3.1).

The AI model achieved near identical performance for male and female patients (AUC 99.1% [95% CI 98.8–99.4] vs. 99.2% [95% CI 98.8–99.5]) and similar performance across all adult age groups (AUC 98.2–100%; Supplementary Table S2).

The expected calibration error (ECE) was 0.005, indicating close agreement between predicted likelihood of disease and actual disease frequency, suggesting that the AI model’s probability estimates are reliable for clinical interpretation. However, this estimate may be optimistic, as model calibration (i.e., agreement between predicted probabilities and observed outcomes) was evaluated on the same validation dataset used to fit the model. To assess the contribution of the underlying multiple instance learning architectures, we compared the performance of the individual CLAM, ABMIL, and TransMIL models with the final ensemble. All architectures demonstrated consistently high performance, with the ensemble providing a small but consistent improvement in discrimination (Supplementary Table S3, Supplementary section S3.2).

We evaluated the AI model, trained exclusively on adult patients, on an independent paediatric dataset comprising duodenal biopsies from 708 unique patients (aged 1–17 years; mean age 13; 49% female) from two hospitals not included in the training data, providing an independent test of generalisability. The test dataset comprised consecutive cases and is representative of routine clinical practice.

On this dataset the AI model achieved an accuracy of 96.2%, sensitivity of 80.2%, and specificity of 98.4%. Restricting analysis to predictions outside the intermediate-probability range (predicted coeliac disease probability <10% or ≥65%; retaining 604/708 [85.3%] of cases), performance improved to 98.8% accuracy, 100% sensitivity, and 98.7% specificity. A detailed review of the seven misclassifications after excluding intermediate-probability predictions is provided in Supplementary Material S3.7. Among high-confidence predictions (defined as “very unlikely” or “very likely” coeliac disease; p < 2% or p ≥ 85%; 467 [66%] of 708 cases retained), no misclassifications were observed (Table 2). Performance improved progressively as analysis was restricted to increasingly confident predictions, demonstrating a favourable trade-off between diagnostic performance and case coverage (Fig. 2B). A detailed summary of retained coeliac disease and non-coeliac disease cases, class-specific coverage, and retained diagnostic errors at each confidence threshold is provided in Supplementary Table S5 in Supplementary Material S3.4.

**Table 2:** Test set results on the paediatric dataset.

	Accuracy	Sensitivity	Specificity	Positive predictive value	Negative predictive value	AUC
Overall (n = 708)	96.2 (94.5–97.4)	80.2 (70.6–87.3)	98.4 (97.1–99.1)	87.3 (78.2–93.0)	97.3 (95.7–98.3)	98.8 (98.2–99.4)
Excluding intermediate-probability predictions (n = 604, 85.3%)	98.8 (97.6–99.4)	100.0 (93.7–100.0)	98.7 (97.4–99.4)	89.1 (79.1–94.6)	100.0 (99.3–100.0)	99.6 (99.3–100.0)
High-confidence predictions (n = 467, 66.0%)	100.0 (99.2–100.0)	100.0 (75.8–100.0)	100.0 (99.2–100.0)	100.0 (75.8–100.0)	100.0 (99.2–100.0)	100.0 (100.0–100.0)
Source						
Edinburgh (n = 237)	94.1 (90.3–96.4)	81.8 (69.7–89.8)	97.8 (94.5–99.1)	91.8 (80.8–96.8)	94.7 (90.5–97.1)	98.4 (97.0–99.7)
Southampton (n = 471)	97.2 (95.3–98.4)	77.4 (60.2–88.6)	98.6 (97.1–99.4)	80.0 (62.7–90.5)	98.4 (96.8–99.2)	99.0 (98.2–99.7)
Sex						
Male (n = 357)	96.9 (94.6–98.3)	79.3 (61.6–90.2)	98.5 (96.5–99.3)	82.1 (64.4–92.1)	98.2 (96.1–99.2)	98.6 (97.5–99.7)
Female (n = 349)	95.4 (92.7–97.2)	80.7 (68.7–88.9)	98.3 (96.1–99.3)	90.2 (79.0–95.7)	96.3 (93.5–97.9)	98.9 (98.2–99.7)
Age, years						
1–5 (n = 77)	98.7 (93.0–99.8)	90.0 (59.6–98.2)	100.0 (94.6–100.0)	100.0 (70.1–100.0)	98.5 (92.1–99.7)	99.4 (98.1–100.0)
6–10 (n = 166)	92.8 (87.8–95.8)	76.9 (57.9–89.0)	95.7 (91.0–98.0)	76.9 (57.9–89.0)	95.7 (91.0–98.0)	97.7 (95.8–99.7)
11–17 (n = 457)	96.9 (94.9–98.2)	80.0 (67.0–88.8)	99.0 (97.5–99.6)	90.9 (78.8–96.4)	97.6 (95.6–98.7)	99.0 (98.3–99.7)

Exclude intermediate-probability predictions leaves cases with a predicted CD probability of <10% or ≥65%. High-confidence predictions include coeliac disease probabilities of <2% or ≥85%. All subgroup analysis is done on all cases including ones within the intermediate-probability range. 95% CIs were calculated for all diagnostic performance metrics using the Wilson score method. AUC = area under the receiver operator characteristic curve.

**Fig. 2:**
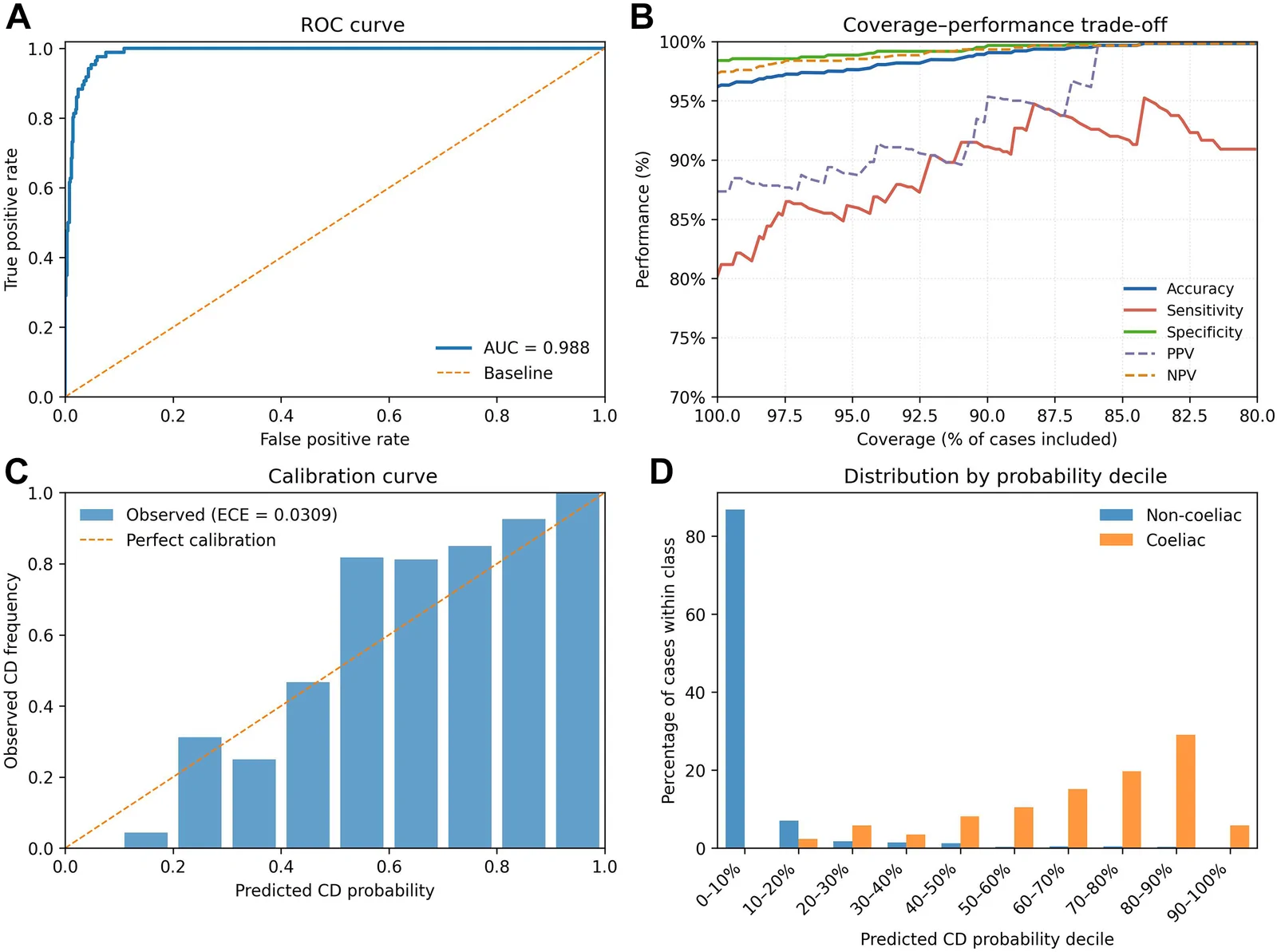
Model discrimination, calibration, and confidence-based performance. (A) ROC curve demonstrating excellent discrimination of coeliac disease (area under the ROC 0.988). (B) Coverage–performance trade-off using symmetric probability thresholds (including cases with predicted probability <*t* or >1−*t*). Restricting analysis to the most confident predictions improved performance, with accuracy approaching ∼99% at approximately 90% coverage. Sensitivity and specificity remained high across the range of coverage. (C) Calibration curve showing good agreement between predicted and observed probabilities (ECE 0.0309), indicating that predicted probabilities are well calibrated. (D) Distribution of predicted probabilities by class, demonstrating separation between coeliac and non-coeliac cases, with most non-coeliac cases assigned low probabilities and coeliac cases concentrated at higher probabilities. CD = coeliac disease. ECE = expected calibration error. ROC = receiver operating characteristic.

Performance was consistent across centres, sexes, and age groups (Table 2), indicating robust generalisability across clinically relevant subgroups. 95% CIs for all diagnostic performance metrics are provided in Supplementary Table S4 in Supplementary Material S3.3. Calibration remained strong in the paediatric cohort, with an expected calibration error of 0.03, indicating close agreement between predicted likelihood and observed disease frequency (Fig. 2). A more detailed assessment of model calibration, including the Brier score, and calibration intercept and slope, is provided in Supplementary Material S3.6.

We next assessed the contribution of biopsy location using paired samples from the first (D1) and second (D2) parts of the duodenum (Fig. 3) in the consecutive subset of the Edinburgh test cohort with paired D1 and D2 biopsies (226 biopsies from 113 patients in the Edinburgh cohort with suspected coeliac disease, of whom 55 (48.7%) were diagnosed with coeliac disease). Diagnostic performance was lower when using either site in isolation (AUC of 87.1% for D2 and 91.8% for D1) than when combining information from both biopsies. When the predicted probabilities of coeliac disease from D1 and D2 were averaged to derive a patient-level prediction, the AUC was 96.6%; when the maximum predicted probability across the two sites was used (as in the primary analysis), the AUC was 95.7%.

**Fig. 3:**
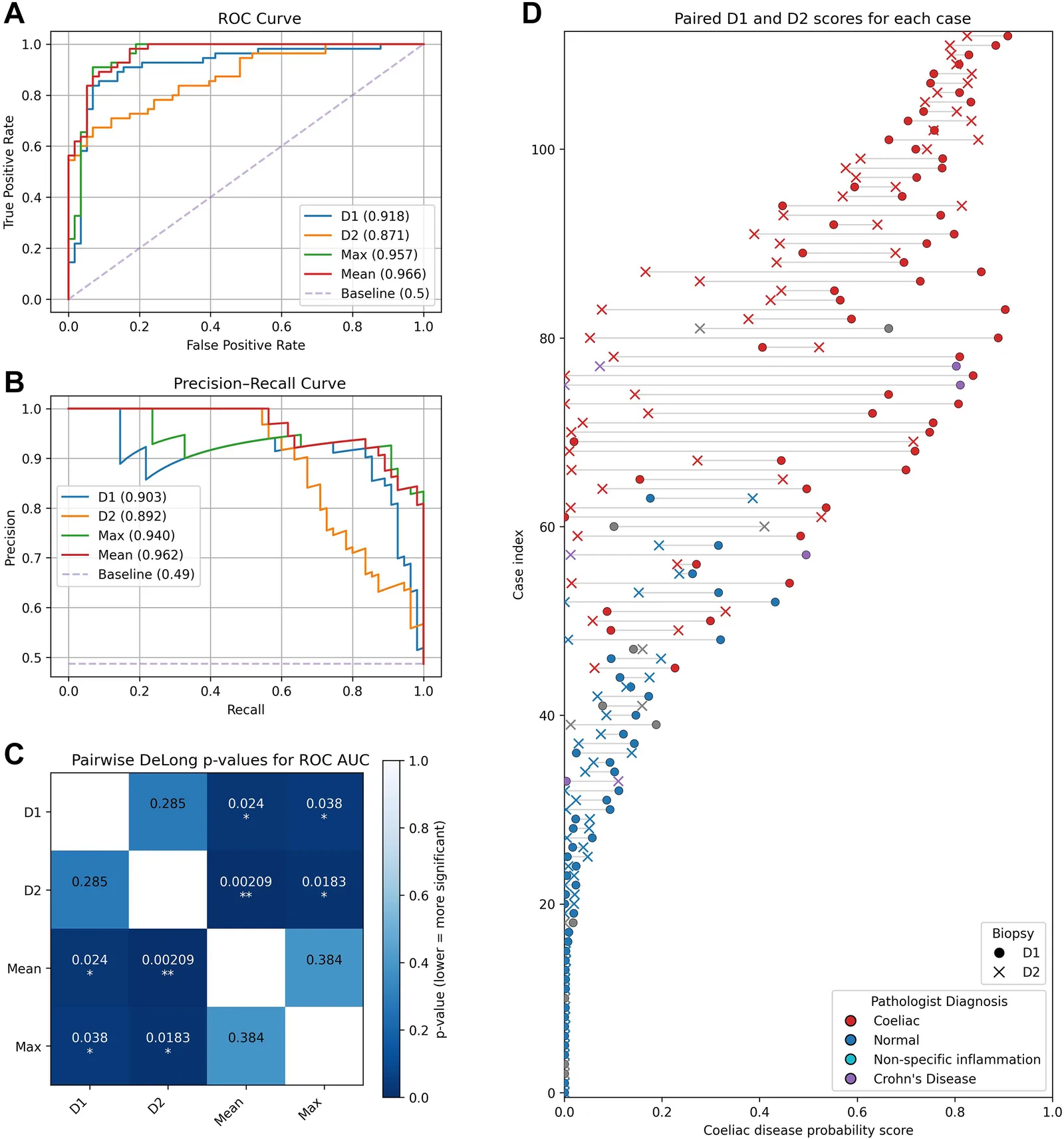
Performance of the AI model on paired D1 and D2 duodenal biopsies. (A) ROC curves for individual biopsy scores (D1 and D2) and combined scores (maximum and mean across biopsies). The mean and maximum scores showed higher discrimination than either biopsy alone. (B) Precision–recall curves for the same models, with the baseline indicating disease prevalence. Performance trends were consistent with ROC analysis. (C) Pairwise comparisons of ROC AUC using DeLong’s test. Lower p values (darker shading) indicate stronger evidence of differences between models. Combined scores were significantly better than D2 and modestly better than D1, with no significant difference between mean and maximum aggregation. (D) Paired comparison of per-patient scores from the two biopsies. Each line represents an individual, linking D1 (circle) and D2 (cross) scores, coloured by histopathological diagnosis. Variation between biopsies within the same patient is evident, and combined scoring reduces this variability. AUC = area under the curve. CD = coeliac disease. ECE = expected calibration error. ROC = receiver operating characteristic.

These differences were statistically significant (DeLong test). For D2, performance was lower than both the mean-based (p < 0.003) and max-based (p < 0.02) combined approaches; similarly, for D1, performance was lower than the mean-based (p < 0.03) and max-based (p < 0.04) approaches. For the prespecified maximum-score aggregation used in the primary analysis, Holm-adjusted p-values^38^ remained statistically significant for comparisons with D1 and D2 (adjusted p = 0.038 and p = 0.037, respectively). This highlights the added diagnostic value of multi-site sampling. Precision–recall analysis demonstrated the same overall pattern as the ROC analysis, with both combined scoring strategies maintaining higher precision across a wide range of recall values than either biopsy site analysed in isolation (Fig. 3B). Analysis of paired predictions further demonstrated substantial intra-patient variability in predicted coeliac disease probability between D1 and D2 samples (Fig. 3D).

## Discussion

In this multicentre study, we found that an AI model trained exclusively on adult duodenal biopsy whole-slide images can generalise effectively to paediatric coeliac disease diagnosis, achieving high accuracy on independent external datasets. Importantly, these datasets incorporated both inter-institutional variation and differences in scanning platforms, providing a test of real-world generalisability. The Edinburgh cohort shared a scanner platform with one of the development centres, whereas the Southampton cohort was digitised using a different platform, reflecting the range of technical settings likely to be encountered during clinical deployment. Performance for each external cohort is reported separately (Table 2), allowing readers to assess model performance independently in each setting.

Despite being trained solely on adult data, the AI model maintained strong performance in children, a population that is frequently underrepresented in medical artificial intelligence development. This finding is particularly relevant given the practical constraints of paediatric data collection, where limited biopsy availability often restricts AI model development. Our results suggest that adult-derived datasets may be sufficient for training robust AI models in coeliac disease, allowing scarce paediatric biopsies to be reserved for independent evaluation and validation rather than training.

Beyond overall diagnostic accuracy, our study emphasises the importance of reliable probability estimates for clinical deployment. In routine practice, diagnostic decisions are rarely binary and instead depend on the degree of certainty associated with a given finding. Modern deep neural networks have been criticised for producing probability estimates that do not reflect true likelihood, for example, assigning very high confidence to incorrect predictions, which limits their clinical utility.^39^ In contrast, our AI model’s predicted probabilities closely matched the observed frequency of disease, meaning that cases assigned a high probability of coeliac disease were very likely to be truly positive, and vice versa. Notably, predictions classified by the AI model as “very likely coeliac disease” or “very unlikely coeliac disease” were all correctly classified within this dataset and accounted for two thirds of cases in the paediatric cohort. This ability of an AI model to provide reliable and clinically interpretable measures of diagnostic confidence is essential for integration into clinical workflows.

Our findings also reinforce the continued importance of established biopsy protocols. Current guidelines from the European Society Paediatric Gastroenterology, Hepatology and Nutrition^26^ and the North American Society For Pediatric Gastroenterology, Hepatology & Nutrition^40^ recommend sampling from both the duodenal bulb (D1) and descending duodenum (D2) to maximise diagnostic yield. This is particularly relevant given the recognised existence of ultra-short coeliac disease, in which villous atrophy is confined to the duodenal bulb, and which may be missed by descending sampling alone.^27^^,^^28^ We demonstrate that this principle remains highly relevant in the context of AI-assisted assessment. AI model performance was significantly improved when both D1 and D2 biopsies were available compared with either site alone, with statistically significant gains in discriminative ability. Furthermore, we observed substantial variability in predicted disease probability between paired D1 and D2 samples within the same patient, underscoring the patchy nature of coeliac disease and the risk of sampling error. These findings suggest that adherence to current biopsy guidelines will be critical for ensuring optimal performance of AI tools in clinical practice and provide independent empirical support for existing recommendations to sample both D1 and D2.

This study has several limitations. First, all data were derived from UK centres; although performance was consistent across multiple institutions and scanner types, generalisability to other populations and health-care settings remains uncertain. In addition, the external paediatric cohorts comprised children undergoing clinically indicated duodenal biopsy for suspected coeliac disease. Accordingly, the findings should be interpreted within this diagnostic context and may not generalise to screening populations. The external paediatric cohorts also differed from the adult training cohort in institution and laboratory/scanner characteristics, meaning the independent effect of patient age on model performance could not be isolated. Furthermore, scanner platform and reference-standard type differed between the two external paediatric cohorts, meaning their independent contributions to the observed between-cohort differences in performance cannot be identified. Second, the reference standard for coeliac disease is imperfect, with recognised interobserver variability in histopathological interpretation. Rigorous multidisciplinary consensus between gastroenterologists and pathologists was used, aiming to improve diagnostic accuracy, but misclassification of a small proportion of biopsies cannot completely be excluded. Only eight cases were resolved through multidisciplinary consensus where this differed from the original histopathological impression, which was too few to meaningfully compare diagnostic performance by reference-standard type; a sensitivity analysis excluding these cases was therefore performed. Third, some subgroup analyses were based on small numbers of coeliac disease cases, particularly in the oldest age group (90–99 years), and should therefore be interpreted with caution. In addition, ethnicity and socioeconomic data were not available, precluding assessment of model performance across these subgroups. Finally, this was a retrospective diagnostic accuracy study, in which direct comparison with paediatric pathologists performance was not performed prospective evaluation within routine clinical workflows will be required before clinical implementation.

Taken together, these findings support the feasibility of deploying AI-based approaches for coeliac disease assessment across age groups and clinical settings. The results also highlight important considerations for safe implementation, including the need for well-calibrated probability outputs (i.e., predicted coeliac disease probabilities that closely reflect the true likelihood of disease) and adequate biopsy sampling (including sampling from both the duodenal bulb (D1) and descending duodenum (D2), in line with current guidelines^26^^,^^40^). High accuracy among high-confidence predictions suggests that a threshold-based approach could enable AI-assisted identification of biopsies that are very unlikely to represent coeliac disease, potentially supporting safe workload reduction in pathology through automated triaging and, following prospective clinical validation, automated reporting of biopsies unlikely to represent coeliac disease.

The clinical importance of accurate and timely diagnosis extends beyond histopathology alone. Increasing evidence demonstrates substantial psychosocial burden among children and adolescents with coeliac disease, including anxiety, social restriction, impaired quality of life, and hypervigilance surrounding gluten exposure.^5^^,^^6^^,^^41^ Prolonged undiagnosed disease is additionally associated with increased long-term morbidity.^42^^,^^43^ Ongoing debate regarding population-based screening programmes is likely to further increase the number of children undergoing confirmatory endoscopic biopsy, driving demand for scalable and reproducible histopathological assessment.

Future work should include prospective evaluation of our AI model in routine diagnostic workflows, direct comparison with pathologist performance, and validation in more diverse international populations. Dedicated investigation of clinically important subgroups, including children with type 1 diabetes, selective IgA deficiency, ultra-short coeliac disease, and screen-detected asymptomatic cases, would help establish whether performance is maintained across the full spectrum of paediatric coeliac disease presentations.

To conclude, we showed in this study that an AI model trained exclusively on adult duodenal biopsy whole-slide images can generalise effectively to paediatric coeliac disease diagnosis across independent clinical centres and scanning platforms. Despite well-documented concerns about the generalisation of adult-trained AI devices to paediatric populations, our findings suggest that key histomorphological features of coeliac disease appear sufficiently conserved to enable robust cross-age generalisation. This has important practical implications, particularly in the context of limited paediatric data availability, and supports a strategy in which paediatric datasets are prioritised for independent validation rather than model training.

Crucially, our AI model provides well-calibrated probability estimates, allowing uncertainty to be communicated in a clinically meaningful way. Within this external test set, all high-confidence predictions were correctly classified and accounted for the majority of cases, underscoring the potential for AI to support decision-making within existing diagnostic pathways. Together with the demonstrated importance of multi-site biopsy sampling, these findings suggest a pathway towards safe, scalable, and clinically integrated AI-assisted assessment of coeliac disease in children.

## Contributors

FJ, PG, MA, and ES conceived and designed the study. FJ developed the AI model, performed all experiments, and drafted the manuscript. KK, SN, JC, and AB oversaw data collection and clinical governance at their respective centres. PG, MA, and ES contributed to subsequent revisions and helped shape the final manuscript. FJ and EJS had access to the study data and verified the study data. All authors reviewed and approved the final version.

## Data sharing statement

The data underlying this study are not publicly available because the ethics approval and participant consent agreements do not permit data sharing.

## Declaration of interests

FJ, MA, ES are all shareholders in Lyzeum.

## Appendix A Supplementary data

Supplementary Sections S1–S4
