# Supplementary material for: Generalisability of an adult-trained AI model to paediatric coeliac disease diagnosis: a multicentre external validation study
Source: eClinicalMedicine. 2026 Oct 12;101:104246. doi: 10.1016/j.eclinm.2026.104246 (PMC13681462; doi:10.1016/j.eclinm.2026.104246)
Supplement: Supplementary file 1 [file mmc1.docx]

# *Generalisability of an Adult-Trained AI Model to Paediatric Coeliac Disease Diagnosis: a Multi-Centre External Validation Study* – Supplementary Materials

# Summary

- Supplementary Section S1 covers the adult training and paediatric test dataset in further detail.
- Supplementary Section S2 describes the methods in more detail.
- Supplementary Section S3 provides further validation and test performance results, including subgroup breakdowns, confidence intervals, MIL architecture comparisons, calibration metrics, and error analysis.
- Supplementary Section S4 describes the software environment used for model development and analysis.

## S1 Dataset

| Diagnosis | Training Dataset | Testing Dataset |
| --- | --- | --- |
| Normal | 6262 | 479 |
| Coeliac Disease | 961 | 86 |
| Chronic / non-specific inflammation | 428 | 103 |
| Adenoma | 182 | 0 |
| Isolated intestinal intraepithelial lymphocytes | 152 | 5 |
| Gastric Heterotopia | 113 | 0 |
| Gastric Metaplasia | 91 | 12 |
| Ulceration | 45 | 0 |
| Carcinoma | 35 | 0 |
| Peptic Duodenitis | 26 | 0 |
| Non-diagnosable | 21 | 8 |
| Lymphangiectasia | 19 | 0 |
| Isolated partial villous atrophy | 17 | 7 |
| Neuroendocrine Tumour | 9 | 0 |
| Transplant | 6 | 0 |
| Brunner Gland Hyperplasia | 5 | 0 |
| Giardia | 5 | 0 |
| Eosinophilic Duodenitis | 5 | 1 |
| Amyloidosis | 1 | 0 |
| Crohn’s Disease | 1 | 6 |
| Other Diagnosis | 37 | 1 |

Table S1. Detailed breakdown of diagnoses of the training and testing dataset.

### S1.1 Adult Training Dataset

The training and validation dataset comprised duodenal biopsy whole-slide images from five UK centres, incorporating both consecutive and enriched cohorts to ensure representation of routine clinical practice alongside sufficient numbers of coeliac disease (CD) and other pathological cases.

At Addenbrooke’s Hospital, 4206 cases were included from two consecutive time periods: 2018–2019 (n=1206) and 2022–2024 (n=3000). These datasets were representative of routine clinical practice and were not enriched for any diagnostic category. The earlier cohort was scanned using the Leica Aperio AT2 scanner, and the later cohort using the Philips IntelliSite Ultra Fast Scanner.

From University Hospital of North Tees, 1705 cases were included, all of which were consecutive cases collected between 2022 and 2024 and scanned using a Hamamatsu NanoZoomer S210 scanner.

From Queen Elizabeth University Hospital, 642 consecutive cases representative of routine clinical practice were included, scanned using the Philips IntelliSite Ultra Fast Scanner.

From University Hospitals Coventry & Warwickshire, 672 cases were included. Of these, 469 were consecutive cases representative of routine clinical practice and scanned using the Philips IntelliSite Ultra Fast Scanner. The remaining cases were selectively enriched to increase representation of coeliac disease and other non-coeliac pathological abnormalities and were scanned using either the Philips IntelliSite Ultra Fast Scanner or Leica Aperio AT2.

From Heartlands Hospital, 400 patients with either normal or coeliac disease diagnoses were included. These were scanned across three platforms (Hamamatsu NanoZoomer XR, Leica Aperio AT2, and Roche Ventana iScan HT), resulting in 1196 whole-slide images.

In total, the training and validation dataset comprised 6262 normal cases, 961 coeliac disease cases, and 1198 cases with other non-coeliac pathological diagnoses. A detailed breakdown of these non-coeliac diagnoses is provided in Table S1.

For the training dataset, reference diagnoses were derived from the original histopathology report. Cases in which the report indicated a high-confidence diagnosis were labelled directly. Where histopathological findings were equivocal or insufficient for confident classification, additional clinical information was reviewed, including coeliac serology and independent assessment by a second pathologist, to establish a reference diagnosis.

### S1.2 Paediatric Testing Dataset

The paediatric test dataset comprised consecutive cases representative of routine clinical practice from two independent centres not included in model training.

From Western General Hospital, Edinburgh, 237 cases were included, scanned using the Philips IntelliSite Ultra Fast Scanner. From University Hospital Southampton, 471 cases were included, scanned using the 3DHISTECH Pannoramic 1000 scanner.

In total, the test dataset comprised 479 normal cases, 86 coeliac disease cases, and 143 cases with other non-coeliac pathological diagnoses. The distribution of these non-coeliac diagnoses differed from the training dataset, reflecting differences in patient age and underlying disease spectrum in paediatric populations. A detailed breakdown is provided in Table S1.

For data from the Southampton cohort, reference diagnoses were taken from the original histopathology report. For cases where the histopathological findings were equivocal, CD serology was also considered. For the Edinburgh cohort, reference diagnoses were established through a multidisciplinary clinical consensus process involving paediatric gastroenterologists and pathologists. This process integrated histopathological findings, serological results, and relevant clinical information, including patient history and endoscopic findings, to determine the final clinical diagnosis. All reference diagnoses were assigned independently of AI model outputs.

## S2 Methods

We performed four-fold leave-one-source-out cross-validation. Data from Glasgow and North Tees were combined due to their low number of coeliac disease (CD) cases.

We trained multiple instance learning (MIL) models, including CLAM, ABMIL, and TransMIL (see Section S3.2) using the implementations described in the original publications. Models were trained for three epochs using the Adam optimizer with a constant learning rate of $2\times{10}^{-4}$ and weight decay of $1\times{10}^{-5}$. The final model from the third training epoch was used for evaluation. All experiments were performed using a fixed random seed (345). Model training was performed on NVIDIA A100-SXM-80GB GPUs.

Stain normalisation was applied using the Macenko method. To improve robustness, feature dropout was employed: for each case, between 20% and 70% of patch-level features were randomly sampled during training. No upper limit was imposed on the number of tissue patches extracted from each whole-slide image.

To address imbalance across data sources and class distributions, we assigned a sampling weight to each training example. For each source, a weight was computed proportional to the square root of its bottleneck class size (corresponding to the number of CD cases). This source-level weight was then evenly distributed across CD-positive and CD-negative samples within that source, ensuring equal contribution within each class. The resulting per-sample weights were normalised and used with a weighted sampling strategy during training to promote balanced learning across both sources and classes.

We trained a post-hoc calibration model to map raw model outputs to well-calibrated probabilities interpretable as absolute risk. Specifically, validation predictions from each individual classifier in the adult dataset were used as inputs to a logistic regression model, which learns a monotonic transformation from uncalibrated scores to predicted probabilities. Calibration was performed separately for each classifier before ensembling.

Because the development dataset was enriched for positive cases (observed prevalence ≈20%) relative to the expected real-world prevalence (10%), inverse probability weighting was applied during calibration. Each observation was assigned a weight proportional to the ratio of the target prevalence to the observed prevalence: positive cases were weighted by $0.1/0.2$, and negative cases by $0.9/0.8$. This reweighting ensures that the calibration model reflects the class distribution expected in clinical practice.

The calibrated model therefore yields probability estimates aligned with real-world event rates, rather than the artificially enriched distribution of the training data. No paediatric test data or labels were used during calibration.

## S3 Validation performance

### S3.1. Detailed Validation Performance by subcategory

The model achieved high validation performance on the adult dataset (n=8421), with an accuracy of 97.7% and AUC of 99.1%. Sensitivity was 89.3% and specificity 98.8%, indicating strong discrimination with high rule-out capability.

Restricting analysis to cases outside the intermediate-probability range (<10% or ≥65%; 94.0% of cases) improved performance (accuracy 98.9%, AUC 99.4), with further gains in high-confidence predictions (<0.02 or >0.85; 85.0% of cases), where sensitivity and specificity reached 98.3% and 99.6%, respectively (AUC 99.6%). Model performance increased with prediction confidence.

Performance was consistent across sites, sexes, and age groups. AUCs exceeded 98.7% across all centres, with some variation in sensitivity (84.6%–93.8%). Performance was similar between females and males, with modest differences in sensitivity (86.6% vs 90.7%). Across age groups, discrimination remained high (AUC 98.2–100%), although sensitivity was lower in older cohorts (e.g., 82.6% in ages 80–90). These findings indicate robust generalisation across diverse subgroups.

|  |  | Accuracy | Sensitivity | Specificity | PPV | NPV | AUC |
| --- | --- | --- | --- | --- | --- | --- | --- |
|  | Overall (n=8421) | 97.7 | 89.3 | 98.8 | 90.4 | 98.6 | 99.1 |
|  | Excluding intermediate-probability predictions (n=7916, 94.0%) | 98.9 | 97.1 | 99.1 | 92.9 | 99.7 | 99.4 |
|  | High-confidence predictions (n=6995, 83.1%) | 99.5 | 98.3 | 99.6 | 95.8 | 99.8 | 99.6 |
| Source | Addenbrookes (n=4206) | 98.1 | 84.6 | 98.8 | 77.6 | 99.2 | 98.7 |
|  | North-Tees (n=1705) | 98.2 | 93.8 | 98.3 | 34.1 | 99.9 | 99.1 |
|  | UHCW (n=672) | 97.9 | 85.4 | 98.7 | 81.4 | 99.0 | 99.1 |
|  | Heartlands (n=1196) | 94.9 | 90.5 | 99.8 | 99.8 | 90.4 | 99.8 |
|  | Glasgow (n=642) | 98.6 | 92.9 | 99.3 | 94.2 | 99.1 | 99.2 |
| Sex | Female (n=4799) | 97.9 | 90.7 | 99.0 | 93.5 | 98.6 | 99.2 |
|  | Male (n=3618) | 97.4 | 86.6 | 98.4 | 84.8 | 98.7 | 99.1 |
| Age | 18-30 (n=590) | 95.3 | 89.7 | 98.0 | 95.6 | 95.1 | 99.2 |
|  | 30-40 (n=717) | 96.2 | 92.1 | 97.7 | 93.6 | 97.2 | 98.8 |
|  | 40-50 (n=1030) | 99.5 | 87.5 | 98.5 | 92.5 | 97.3 | 98.2 |
|  | 50-60 (n=1335) | 98.5 | 91.4 | 99.2 | 91.4 | 99.2 | 99.2 |
|  | 60-70 (n=1764) | 98.1 | 88.4 | 99.0 | 89.0 | 98.9 | 99.5 |
|  | 70-80 (n=1921) | 98.3 | 87.8 | 98.7 | 75.8 | 99.5 | 99.1 |
|  | 80-90 (n=1000) | 98.5 | 82.6 | 99.3 | 84.4 | 99.2 | 99.7 |
|  | 90-100 (n=64) | 96.9 | 100.0 | 96.8 | 33.0 | 100.0 | 100.0 |

Table S2. Leave-one-source-out Cross-Validation Performance. Excluding intermediate probability range predictions meant keeping cases with a predicted CD probability <10% or ≥65%; High-confidence predictions include predicted CD probabilities of <2% or ≥85%. All subgroup analysis is done on all cases including ones within the intermediate probability.

### S3.2. Validation performance – MIL comparison

|  | All cases | | Excluding intermediate-probability cases | | Very confident cases | |
| --- | --- | --- | --- | --- | --- | --- |
|  | percentage of total cases | AUC | percentage of total cases | AUC | percentage of total cases | AUC |
| CLAM | 100 | 98.7 | 93.9 | 99.0 | 83.1 | 99.2 |
| ABMIL | 100 | 99.1 | 94.5 | 99.3 | 84.4 | 99.6 |
| TransMIL | 100 | 98.8 | 93.3 | 99.1 | 84.9 | 99.2 |
| MIL-Ensemble | 100 | 99.1 | 94.0 | 99.4 | 85.0 | 99.6 |

Table S3. We compare performance across different MIL architectures. Performance is similar across all three MIL types.

We compared the performance of three multiple instance learning (MIL) architectures, CLAM, ABMIL, and TransMIL, and an ensemble combining all three approaches. To ensure a fair comparison, each MIL model was trained using three independent random seeds, and performance was averaged across runs. The ensemble was constructed by combining one trained model from each architecture and aggregating their predictions.

All models demonstrated high discrimination across evaluation settings. On the full dataset, AUCs ranged from 98.7% (CLAM, TransMIL) to 99.1% (ABMIL and ensemble). Performance improved when excluding intermediate-probability predictions, with AUCs increasing to 99.0–99.4%. Among very high-confidence cases, all models achieved excellent discrimination (AUC ≥99.2%), with ABMIL and the ensemble reaching the highest performance (99.6%).

Overall, differences between individual MIL architectures were modest, with consistently high performance across all methods. The ensemble provided a small but consistent improvement in discrimination, particularly in the high-confidence subset, suggesting complementary strengths across model types.

## S3.3 Further Test Performance – Confidence Intervals

|  |  | Accuracy | Sensitivity | Specificity | PPV | NPV | AUC |
| --- | --- | --- | --- | --- | --- | --- | --- |
|  | Overall (n=708) | 96.2 (94.5-97.4) | 80.2 (70.6-87.3) | 98.4 (97.1-99.1) | 87.3 (78.2-93.0) | 97.3 (95.7-98.3) | 98.8 (98.2-99.4) |
|  | Excluding intermediate-probability predictions (n=604, 85.3%) | 98.8 (97.6-99.4) | 100.0 (93.7-100.0) | 98.7 (97.4-99.4) | 89.1 (79.1-94.6) | 100.0 (99.3-100.0) | 99.6 (99.3-100.0) |
|  | High-confidence predictions (n=467, 66.0%) | 100.0 (99.2-100.0) | 100.0 (75.8-100.0) | 100.0 (99.2-100.0) | 100.0 (75.8-100.0) | 100.0 (99.2-100.0) | 100.0 (100.0-100.0) |
| Source | Edinburgh (n=237) | 94.1 (90.3-96.4) | 81.8 (69.7-89.8) | 97.8 (94.5-99.1) | 91.8 (80.8-96.8) | 94.7 (90.5-97.1) | 98.4 (97.0-99.7) |
|  | Southampton (n=471) | 97.2  (95.3-98.4) | 77.4 (60.2-88.6) | 98.6 (97.1-99.4) | 80.0 (62.7-90.5) | 98.4 (96.8-99.2) | 99.0 (98.2-99.7) |
| Sex | Male (n=357) | 96.9 (94.6-98.3) | 79.3 (61.6-90.2) | 98.5 (96.5-99.3) | 82.1 (64.4-92.1) | 98.2 (96.1-99.2) | 98.6 (97.5-99.7) |
|  | Female (n=349) | 95.4 (92.7-97.2) | 80.7 (68.7-88.9) | 98.3 (96.1-99.3) | 90.2 (79.0-95.7) | 96.3 (93.5-97.9) | 98.9 (98.2-99.7) |
| Age | 1-5 (n=77) | 98.7 (93.0-99.8) | 90.0 (59.6-98.2) | 100.0 (94.6-100.0) | 100.0 (70.1-100.0) | 98.5 (92.1-99.7) | 99.4 (98.1-100.0) |
|  | 6-10 (n=166) | 92.8 (87.8-95.8) | 76.9 (57.9-89.0) | 95.7 (91.0-98.0) | 76.9 (57.9-89.0) | 95.7 (91.0-98.0) | 97.7 (95.8-99.7) |
|  | 11-17 (n=457) | 96.9 (94.9-98.2) | 80.0 (67.0-88.8) | 99.0 (97.5-99.6) | 90.9 (78.8-96.4) | 97.6 (95.6-98.7) | 99.0 (98.3-99.7) |

Table S4. 95% confidence intervals for the test performance.

To quantify the uncertainty associated with the reported performance estimates, 95% confidence intervals (CIs) were calculated for all diagnostic performance metrics. Confidence intervals for accuracy, sensitivity, specificity, positive predictive value (PPV), and negative predictive value (NPV) were calculated using the Wilson score method, which provides reliable interval estimates for binomial proportions, including when observed performance is close to 0% or 100%. Confidence intervals for the area under the receiver operating characteristic curve (AUC) were calculated using DeLong's non-parametric method. These confidence intervals reflect the uncertainty arising from the finite size of the evaluation cohorts. Wider confidence intervals observed in smaller subgroups and high-confidence prediction subsets reflect the smaller number of evaluable cases rather than reduced model performance.

## S3.4 Further Test Performance – Confidence Levels

|  | CD retained | Non-CD retained | CD coverage | Non-CD coverage | Errors retained |
| --- | --- | --- | --- | --- | --- |
| All | 86 | 622 | 100% | 100 | 17 |
| Excluding intermediate-probability predictions | 57 | 547 | 66% | 88 | 7 |
| High-confidence predictions | 12 | 455 | 14 | 73 | 0 |

Table S5. Selective classification performance at different confidence thresholds. Numbers of retained coeliac disease (CD) and non-CD cases, class-specific coverage, and retained diagnostic errors for the full cohort, excluding intermediate-probability predictions, and the high-confidence subset.

Table S5 summarises the number of CD and non-CD cases retained at each confidence threshold together with class-specific coverage and the number of retained errors. Increasing the confidence threshold reduced the proportion of retained cases, particularly among CD cases, but progressively reduced the number of diagnostic errors from 17 across the full cohort to 7 after excluding intermediate-probability predictions and to 0 within the high-confidence subset. These findings illustrate the trade-off between diagnostic coverage and performance when restricting predictions to increasingly confident cases.

### S3.5 Further Test Performance – Uncertain Histopathological Diagnosis

To assess the potential impact of the multidisciplinary consensus reference standard used in the Edinburgh cohort, we performed a sensitivity analysis excluding the eight cases in which the original histopathology report described the biopsy as *suspicious for coeliac disease* and the final multidisciplinary diagnosis was coeliac disease. No definitive histopathological diagnosis was revised by the multidisciplinary review.

Excluding these eight equivocal cases resulted in only minor changes to model performance. Accuracy increased from 94.1% to 95.2%, sensitivity from 81.8% to 85.4%, and the negative predictive value from 94.7% to 96.2%, while specificity remained unchanged at 97.8%. The positive predictive value changed marginally from 91.8% to 91.1%, and the area under the receiver operating characteristic curve remained essentially unchanged (0.984 vs. 0.985). These findings indicate that the small number of cases requiring multidisciplinary consensus had minimal impact on the overall performance estimates for the Edinburgh cohort.

Notably, all eight cases were assigned either intermediate (10%≤p<65%) or likely (65%≤p<85%) coeliac disease probabilities by the AI (range 0.23-0.81), consistent with the diagnostic uncertainty reflected in the original histopathology reports.

### S3.6 Further Test Performance – Calibration Performance

Calibration on the paediatric test set was further assessed using complementary measures including the Brier score, expected calibration error (ECE), and logistic recalibration statistics. The Brier score measures the overall accuracy of probabilistic predictions, with lower values indicating better agreement between predicted probabilities and observed outcomes. The ECE summarises the average absolute difference between predicted probabilities and observed event frequencies across 10 equally spaced probability bins, with lower values indicating better calibration. The Brier score was 0.0308 and the ECE was 0.0309, consistent with excellent overall probabilistic performance and good agreement between predicted probabilities and observed outcome frequencies. Logistic recalibration yielded a calibration intercept of 0.27 (95% CI: −0.19 to 0.73), indicating no evidence of systematic over- or underprediction, and a calibration slope of 1.73 (95% CI: 1.33 to 2.14). A calibration slope greater than 1 suggests that predicted probabilities were relatively conservative, with estimates compressed towards intermediate probabilities rather than being overly extreme. Such conservative probability estimates may be advantageous in clinical decision support, where expressing uncertainty for equivocal cases can facilitate appropriate clinical review rather than conveying unwarranted confidence. Calibration-in-the-large was 0.0001 (95% CI: −0.34 to 0.34), indicating negligible overall prediction bias. These findings were consistent with the visual assessment of the calibration curve shown in Figure 2.

### S3.7 Further Test Performance – AI Error Analysis following Exclusion of intermediate-probability Predictions

We reviewed the seven misclassifications remaining after excluding intermediate-probability predictions. All seven were false-positive predictions. Errors occurred across both external centres (Southampton, *n*=4; Edinburgh, *n*=3), with no obvious clustering by age (6–10 years, *n*=5; 11–17 years, *n*=2) or sex (female, *n*=4; male, *n*=3). Three cases had positive coeliac serology despite alternative or non-diagnostic histopathological findings. Histopathological diagnoses comprised chronic non-specific inflammation (*n*=3), Crohn's disease (*n*=2), isolated intraepithelial lymphocytosis (*n*=1), and one non-diagnostic biopsy. These findings suggest that the residual misclassifications predominantly occurred in cases with overlapping clinical or histopathological features. None of the residual misclassifications occurred within the high-confidence prediction group (>85% predicted probability), indicating that the model's most confident predictions remained correctly classified.

## S4 Software environment

All image processing, model development, statistical analysis, and figure generation were performed using open-source software in Python (version 3.11). Whole-slide image handling and patch extraction were performed using OpenSlide (v1.3.1), image processing using OpenCV (v4.8.1.78), scikit-image (v0.25.2), Pillow (v12.0.0), and NumPy (v1.24.4). Feature extraction using the Hibou-b foundation model was implemented using PyTorch (v2.9.11+cu128), Torchvision (v0.24.1+cu128), and the Hugging Face Transformers library (v4.57.3). Data handling was performed using pandas (v2.1.0). Statistical analyses, including performance metric calculations and model calibration, were performed using scikit-learn (v0.25.2). Model serialisation was performed using the Python pickle module, and figures were generated using Matplotlib (3.7.2.).

All software packages used are open source and are widely adopted within the machine learning and scientific computing communities.
